# Relationship between Nutritional Status and Clinical Outcome in Patients Treated for Lung Cancer

**DOI:** 10.3390/nu13103332

**Published:** 2021-09-23

**Authors:** Jacek Polański, Mariusz Chabowski, Natalia Świątoniowska-Lonc, Krzysztof Dudek, Beata Jankowska-Polańska, Jan Zabierowski, Grzegorz Mazur

**Affiliations:** 1 Department of Internal Medicine, Occupational Diseases, Hypertension and Clinical Oncology, Wrocław Medical University, 50556 Wrocław, Poland; polanoo@hotmail.com (J.P.); grzegorz.mazur@umed.wroc.pl (G.M.); 2 Department of Oncology and Palliative Care, Wrocław Medical University, 51618 Wrocław, Poland; 3 Department of Clinical Nursing, Faculty of Health Science, Wrocław Medical University, 51618 Wrocław, Poland; natalia.swiat@o2.pl (N.Ś.-L.); bianko@poczta.onet.pl (B.J.-P.); 4 Faculty of Mechanical Engineering, Technical University of Wrocław, 50370 Wrocław, Poland; krzysztof.dudek@pwr.edu.pl; 5 Student Research Group No 180, Faculty of Medicine, Wrocław Medical University, 50367 Wrocław, Poland; jan.zabierowski@student.umed.wroc.pl

**Keywords:** nutrition, lung cancer, clinical outcome, quality of life, survival

## Abstract

Background. Between 34.5% and 69% of the patients with lung cancer are at risk of malnutrition. Quality of life (QoL) and physical status assessment provides valuable prognostic data on lung cancer patients. Malnutrition is a prognostic parameter for clinical outcome. Therefore, the identification of significant factors affecting the clinical outcome and QoL is important. The purpose of this study was to evaluate the relationship between nutritional status and outcome, i.e., overall survival, time to tumor progression, and QoL, in lung cancer patients. Materials and methods. We performed a systematic search of the Pubmed/MEDLINE databases per the Cochrane guidelines to conduct a meta-analysis consistent with the PRISMA statement, using the following keywords: “lung cancer,” “malnutrition,” “nutrition,” “quality of life,” “well-being,” “health-related quality of life,” and “outcome.” Out of the 96 papers identified, 12 were included in our meta-analysis. Results. Our meta-analysis shows that patients with a good nutritional status have a better QoL than malnourished patients in the following functioning domains: physical (*g* = 1.22, 95% CI = 1.19 to 1.46, *p* < 0.001), role (*g* = 1.45, 95% CI = 1.31 to 1.59, *p* < 0.001), emotional (*g* = 1.10, 95% CI = 0.97 to 1.24, *p* < 0.001), cognitive (*g* = 0.91, 95% CI = 0.76 to 1.06, *p* < 0.001), and social (*g* = 1.41, 95% CI = 1.27 to 1.56, *p* < 0.001). The risk of death was significantly higher in malnourished than in well-nourished patients (*HR* = 1.53, 95% CI = 1.25 to 1.86, *p* < 0.001). Nutritional status was significantly associated with survival, indicating that patients with a poorer nutritional status are at more risk of relapse. Conclusions. Nutritional status is a significant clinical and prognostic parameter in the assessment of lung cancer treatment. Malnutrition is associated with poorer outcome in terms of overall survival, time to tumor progression, and QoL in patients treated for lung cancer.

## 1. Introduction

Lung cancer is the leading cause of cancer-related death in Europe and worldwide [1]. The treatment of cancer, and in particular, non-small-cell lung carcinoma (NSCLC) has evolved in the last few years. Patients with metastatic NSCLS can now receive treatment tailored to the specific alterations and mutations identified in the genes or proteins of their cancer [2]. These treatment options are associated with better response to treatment and longer survival, compared to the previous standard based on chemotherapy [3]. Despite these advances, however, the median overall survival for patients with metastatic NSCLC is still less than 1 year [3], and less than half of the patients see a significant decrease in their tumor burden with immunotherapy alone [4]. Researchers are constantly seeking to identify factors associated with treatment effectiveness and better response to immunotherapy. Among these factors, patient age, comorbidities, nutritional status, and weight loss during or before treatment have been proposed [5]. Published findings indicate that malnutrition and risk of malnutrition are correlated with time to tumor progression and overall survival [6,7,8,9,10,11,12,13,14]. Moreover, the cytokine IL-8 (interleukin 8) may be linked to cachexia [15].

Between 34.5% and 69% of the patients with lung cancer are at risk of malnutrition [16]. Malnutrition and, even more so, cachexia have been described as prognostic outcome parameters associated with poorer prognosis; lower treatment effectiveness due to poorer treatment tolerance, higher treatment cost, and more frequent hospitalizations; shorter survival; and poor QoL [5,6,7,8,9,10,11,12,13,14,17,18,19]. Unfortunately, even though hospitalized patients undergo obligatory nutritional assessment, malnutrition, sarcopenia, and cachexia still often go undiagnosed and untreated [20]. Patients with advanced cancers and those who have had multiple hospitalizations are most at risk. Notably, the treatment itself often affects patients’ nutritional status by causing symptoms such as appetite loss, dysphagia, nausea, vomiting, diarrhea, and ulcerations of the oral or intestinal mucosa [21]. 

The diagnosis of nutritional disorders in patients with lung cancer is not sufficient. It is equally important to differentiate between body weight and body composition problems. Malnutrition may lead to poorer physical and mental functioning and significantly affect patients’ clinical condition [22]. Cancer-related malnutrition is associated with metabolic disorders, which may not respond to nutrient supplementation [16]. Cachexia manifests with rapid weight loss, appetite dysfunction, and early satiety [16]. It is also accompanied by general weakness, fatigue, weakened immunity, and poor overall condition. Patients may experience poorer physical performance, difficulties in daily activities, and reduced mobility, resulting in more dependence on others, difficulties in family and social life, lower mood, and feelings of loneliness and isolation. Lung cancer-related skeletal muscle wasting (sarcopenia) has been linked to shorter survival, reduced tolerance to chemotherapy, decreased QoL, and diminished functional ability [22]. In lung cancer patients, nutritional deficiencies are the result of insufficient calorie intake [20]. Nutritional status assessment and nutritional interventions must be included as an integral part of treatment in the lung cancer patient population. 

Researchers have described an adverse association of weight loss, both before and after diagnosis, with shorter survival and a higher risk of death; conversely, there is evidence of a beneficial relationship between higher body weight (with a BMI > 23) and better outcome, including longer survival, in lung cancer patients [23]. A strong correlation between weight loss and QoL in lung cancer patients has also been demonstrated [5], but no clear evidence is available to guide strategies for the implementation of integrated nutritional care standards that would provide comprehensive benefits in terms of improving treatment effectiveness, patient functioning, and QoL [5]. The literature often focuses on cancer patients’ nutritional status or their QoL and links one of the two with the clinical aspects of treatment. However, papers linking all these elements and evaluating their interrelationships remain scarce. 

Quality of life (QoL) and physical status assessment provides valuable prognostic data on lung cancer patients. The identification of factors significant for a patient, affecting their reported QoL, is extremely important. Therefore, the purpose of this study was to evaluate the relationship between nutritional status and outcome in lung cancer patients. Following previous publications, we defined outcome as including QoL, mortality, disability, and time of hospitalization [6,7,8,9,10,11,12,13,14,17,18,19].

## 2. Materials and Methods

### 2.1. Search Strategies

**Figure 1:**
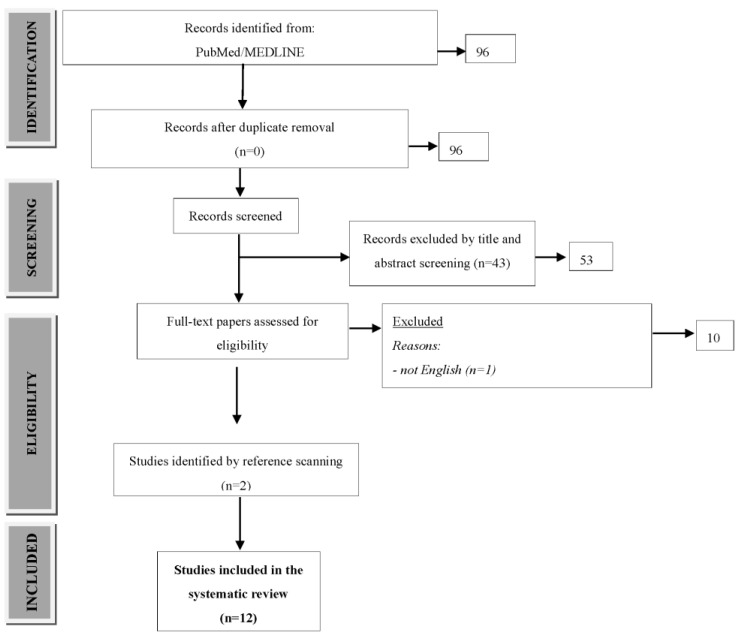
Study flow diagram.

We performed a systematic search of the Pubmed/MEDLINE databases per the Cochrane guidelines to conduct a meta-analysis consistent with the PRISMA (Preferred Reporting Items for Systematic review and Meta-Analysis) statement (Figure 1) [24].

First, we identified all published studies that addressed the relationship between nutritional status and outcome in patients treated for lung cancer and used the terms (lung cancer [Title/Abstract] AND (malnutrition [Title/Abstract] OR nutrition [Title/Abstract] AND (quality of life [Title/Abstract] OR well-being [Title/Abstract] OR health-related quality of life [Title/Abstract] OR outcome), yielding 246 papers. The search limits were defined as “English” (language), “1 January 2000,” and “31 December 2021” (publication date), Adult +19, and full text (96).

The exclusion criteria were as follows: review articles, meta-analyses, case studies, study protocols, no numerical data, no assessment of outcome, and duplicates. Subsequently, three reviewers (JP, NŚL, and MC) selected relevant studies for inclusion by examining the remaining titles, abstracts, or full papers (n = 12). Their analysis considered publication bias, selective reporting of research, and duplication of publications. To ascertain the validity of eligible randomized trials, reviewers were working independently to reliably determine the adequacy of randomization and concealment of allocation, blinding of patients, data collectors, and outcome assessors. Disagreements were resolved by consensus discussion. The meta-analyses were performed by computing relative risks (RRs) using the random effects model. Quantitative analyses were performed on an intention-to-treat basis and were confined to data derived from the follow-up period. RRs and 95% confidence intervals for each type of intervention were calculated.

At the first stage, all records were identified from searches of the electronic databases. At the second stage, three researchers (JP, NŚL, and MC) independently screened the titles and abstracts to identify potentially eligible studies and remove duplicates. At the third stage, studies that were potentially eligible were selected for full-text review. Disagreements were resolved by consensus discussion. Ultimately, 12 full-text papers were included in subsequent statistical analyses (Figure 1 and Table 1). We made every effort to include all primary studies meeting the adopted criteria in our review. The quality of the primary studies was assessed, and the extent to which the studies met the reliability criteria, if at all, was determined. The process of selecting and evaluating primary studies was repeated.

### 2.2. Description of the Included Studies

The 12 studies included in the meta-analysis [6,7,8,9,10,11,12,13,14,17,18,19] were performed in 9 countries in 4 continents. The meta-analysis included research papers published in English in the years 2000–2021 in one of the specified databases. Studies on children, other meta-analyses, review articles, study protocols, duplicates, and studies with incomplete data were excluded from the meta-analysis. 

In the analyzed studies, patient inclusion criteria were as follows: written informed consent (4 studies); lung cancer diagnosis confirmed by histopathological examination (5 studies); age above 18 years (4 studies), understanding the questionnaire items (1 study); patients with stomach, colon, lung, esophageal, liver, or pancreaticobiliary (pancreas, common bile duct, ampulla of Vater, and gallbladder) cancer (1 study); age between 20 and 80 years (1 study); hemoglobin level >9.0 g/dL (1 study); absolute neutrophil count >1500/mm^3^ (1 study); platelet count >100,000/mm^3^ (1 study); total bilirubin <3.0 mg/dL (1 study), creatinine <1.5 mg/dL (1 study); Eastern Cooperative Oncology Group (ECOG) score of ≤2 (3 studies); eligibility to receive paclitaxel (175 mg/m^2^) and cisplatin (80 mg/m^2^) as first-line palliative chemotherapy every 3 weeks for at least two and a maximum of six cycles (1 study); treatment with cisplatin-based chemotherapy and concurrent thoracic radiotherapy followed by surgical resection (1 study); pathologically proven mediastinal lymph node involvement (N2 or N3 disease), by transbronchial fine needle aspiration and/or by esophageal ultrasonography (endoscopic ultrasound-guided fine needle aspiration) or mediastinoscopy (1 study); superior sulcus tumor (SST; 1 study); tumor stage cT4 on the basis of a combination of clinical signs (e.g., neurological) and/or imaging studies such as computed tomography scan or magnetic resonance imaging (e.g., involvement of vertebra; 1 study); ability to tolerate cisplatin-based chemotherapy (1 study); measurable, non-irradiated disease according to the Response Evaluation Criteria in Solid Tumors (RECIST; 1 study); adequate functional reserve of the major organ systems (1 study).

Patient exclusion criteria were as follows: uncertain cancer diagnosis (1 study); lack of consent to participate in the study (2 studies); coexistence of other malignant tumors (1 study); heart failure exacerbation (2 studies); severe chronic obstructive pulmonary disease (1 study); asthmatic condition (1 study); hemodynamic instability (1 study); cognitive impairments (1 study); unstable angina or myocardial infarction within the past 6 months (1 study); significant arrhythmias requiring medication (1 study); conduction abnormalities such as greater than second-degree atrioventricular block (1 study); uncontrolled hypertension (1 study); liver cirrhosis (child class B and C; 2 studies); interstitial pneumonia (1 study); pulmonary adenomatosis (1 study); psychiatric disorders that may interfere with protocol compliance (1 study); unstable diabetes mellitus (1 study); uncontrolled ascites or pleural effusions as well as active infections (4 studies); poor functional performance status (1 study); previous treatment (surgical, radiotherapy, and/or chemotherapy; 1 study); a history of previous malignancies (other than non-melanoma skin tumors) within the last 5 years (2 studies); severe comorbid condition(s) (1 study); anti-inflammatory treatment (2 studies); chronic diseases (i.e., chronic renal failure; 1 study); broncho-esophageal fistula without an esophageal obstruction (1 study); stricture due to radiotherapy (1 study); patients who underwent the procedure because of an impaired swallowing function itself, such as central nervous systemic, oropharyngeal or transient postoperative problems (1 study); patients who underwent the procedure previously at another hospital (1 study); and malignant dysphagia due to other primary cancers (1 study).

### 2.3. Data Extraction

An initial database was developed, pilot-tested, and refined to ensure consistency with the outcomes reported in the literature. Data were independently extracted from eligible articles by three reviewers. Data extraction discrepancies between the reviewers were resolved by consensus.

The following information was extracted from each included trial: (1) characteristics of trial participants (including age, gender, stage, and severity of disease), the trial’s inclusion and exclusion criteria; (2) type of intervention; (3) type of outcome measure. Risk of bias was established using the Newcastle–Ottawa scale. 

### 2.4. Methods

#### 2.4.1. Questionnaires Used in the Studies

In the analyzed papers, the patients’ nutritional status was evaluated using the MNA and SGA questionnaires and BMI and albumin measurements (Table 2).

#### 2.4.2. QoL Questionnaires

- The EORTC QLQ-C30 questionnaire allows for a comprehensive analysis of a patient’s perceived health and functioning in the physical, emotional, and social dimensions. It comprises 30 items in 5 functional scales (physical, role, emotional, cognitive, and social functioning); 3 symptom scales: fatigue, nausea and vomiting, and pain; and 6 single items for recording the severity of shortness of breath, insomnia, appetite loss, constipation, diarrhea, and financial difficulties. The last two items are used to globally evaluate a respondent’s health [12,17,18,19,25].
- The QLQ-LC13 is a lung-cancer-specific module comprising 13 items on specific symptoms such as dyspnea, coughing, hemoptysis, localized pain, adverse effects of treatment (mouth or tongue pain, dysphagia, neuropathy, and hair loss), and treatment-related pain. The results are converted to a 0–100 scoring range, with higher scores indicating more severe symptoms [17,26].

#### 2.4.3. Nutritional Status Assessment Questionnaires and Clinical Parameters

- The MNA comprises two parts: an initial screening (MNA—short form, or MNA-SF) and a more comprehensive assessment part (full MNA). The first part concerns food intake reduction, weight loss, and severe disease within the 3 months preceding the assessment, as well as the patient’s BMI and mobility. The maximum score is 14 points. The second part, patient assessment, records the mode of feeding, the intake of specific diet components, and medication, as well as the measured mid-arm and calf circumferences. The maximum score for this part is 16 points. The sum total of the scores from both parts represents the “malnutrition indicator score,” with a maximum of 30 points. The authors have suggested three nutritional status categories: “normal nutritional status” at 24–30 points, “at risk of malnutrition” at 17–23 points, and “malnourished” below 17 points. Validation studies have demonstrated high reliability and validity of the instrument (scale sensitivity—97.9%; scale specificity—100%) [10,11,17,27].
- Subjective global assessment (SGA) is commonly used as a self-reported assessment tool to evaluate the nutritional status of patients with cancer on the basis of weight loss, food intake, and symptoms. Accordingly, patients are classified as well-nourished (category A) or malnourished (categories B + C) based on their total PG-SGA scores [6,8,19,28].
- AC/S—a score of ≤24 in the AC/S scale—would be sufficient to establish a diagnosis of anorexia [6,8,12,29].
- Albumin is a liver protein found in the blood serum with a half-life of 14–20 days. It is a carrier of various mineral components, hormones, and fatty acids and helps maintain oncotic pressure in the capillaries. It has been used as a marker for malnutrition for decades. The reference range in albumin testing is 3.5–5.5 g/dL or 35–55 g/L [7,8,10,30].

### 2.5. Statistical Analysis

Our meta-analysis was performed using the Statistica 13.3 software (TIBCO Software Inc., Palo Alto, CA, USA). The heterogeneity of the primary study results was assessed using the Q statistic based on χ^2^ and its associated *p*-value. If the heterogeneity test result was significant (*p* < 0.1), the meta-analysis was performed using the random effects model. For *p* > 0.1, the meta-analysis relied on the fixed effects model. The percentage of heterogeneity between study estimates was determined using the I^2^ statistic. 

For differences in QoL between patients in different nutritional status groups, the effect size was measured based on the corrected standardized mean difference—Hedge’s g and its 95% confidence interval (CI). The statistic was calculated based on the information on mean values, their distribution, and sample size in groups.

For overall survival (OS) and time to tumor progression (TTP), we used hazard ratios (HR) calculated using the Cox regression coefficient. 

Any publication bias was estimated using Egger’s test. We also performed sensitivity analysis using the trim-and-fill method and funnel plot symmetry analysis to detect the impact of publication bias on summary results. Findings at *p* < 0.05 were considered statistically significant.

## 3. Results

### 3.1. Impact of Nutritional Status on Outcome in Lung Cancer Patients

The systematic review and meta-analysis looked at selected QoL domains, overall survival (OS), and time to tumor progression (TTP) in the population of lung cancer patients broken down by nutritional status. The premise of the review also included the significance of nutritional status for disability, rehospitalization, and mortality in lung cancer patients, but no papers addressing all these endpoints were found.

In the analyzed papers, patients’ nutritional status was evaluated using questionnaires (MNA or SGA), or measures such as BMI, weight loss (WL), albumin levels, and anorexia or cachexia. Due to the different types of information available on patients’ nutritional status, we adopted a dichotomous classification into well-nourished and malnourished patients. The malnourished group included categories B and C, i.e., at risk of malnutrition or malnourished in the MNA and moderately or severely malnourished in the SGA; underweight patients (BMI < 18 kg/m^2^); patients with involuntary weight loss >5%; with albumin levels <3.5 mg/dL; and with anorexia (A/SC ≤ 32; *n* = 737).

QoL in six domains was evaluated using the EORTC QLQ-C30 or FAACT questionnaire.

### 3.2. QoL

**Figure 2:**
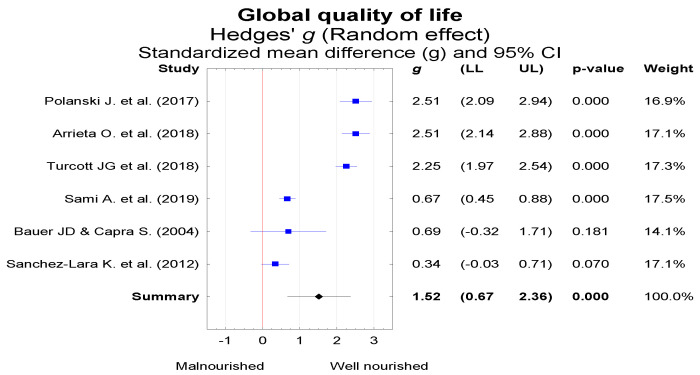
Global QoL in lung cancer patients with a different nutritional status (expressed as corrected standardized mean difference—Hedges’ g). LL—lower limit of the confidence interval, UL—upper limit of the confidence interval for the effect measure (*g*).

QoL was assessed in a group of 289 patients with normal nutritional status and 737 patients who were malnourished or at risk of malnutrition. The mean patient age was 63.1 ± 2.1 years. A total of 56.7% of the studied patients were male. QoL in selected domains for the patients differing by nutritional status was evaluated using the EORTC QLQ-C30 or FAACT questionnaire. Global QoL was assessed in six studies (Figure 2). In these six studies, clear heterogeneity was identified (Q = 173.2, df = 5, *p* < 0.001; I^2^ = 97.1%), and therefore, the random effects model was used for the analysis. Summary results suggest a significant difference in global QoL between well-nourished and malnourished patients (*g* = 1.52, 95% CI = 0.67 to 2.36, *p*< 0.001). 

**Figure 3:**
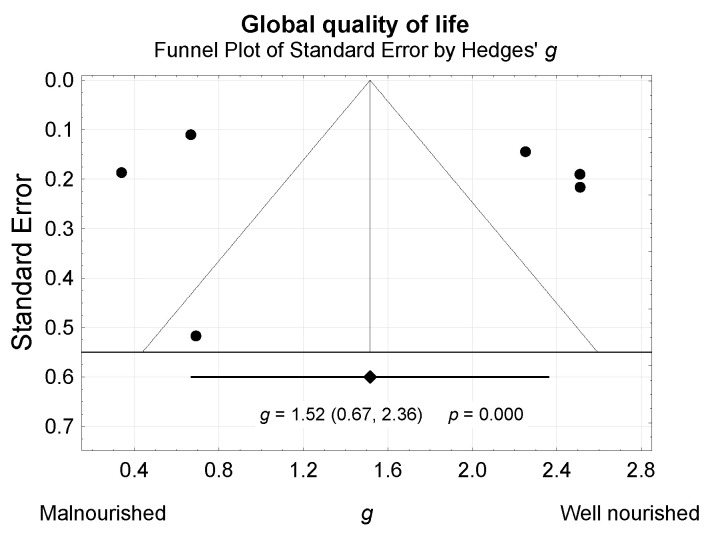
Funnel plot for visual assessment of the probability of systematic bias due to selective publication of studies.

**Figure 4:**
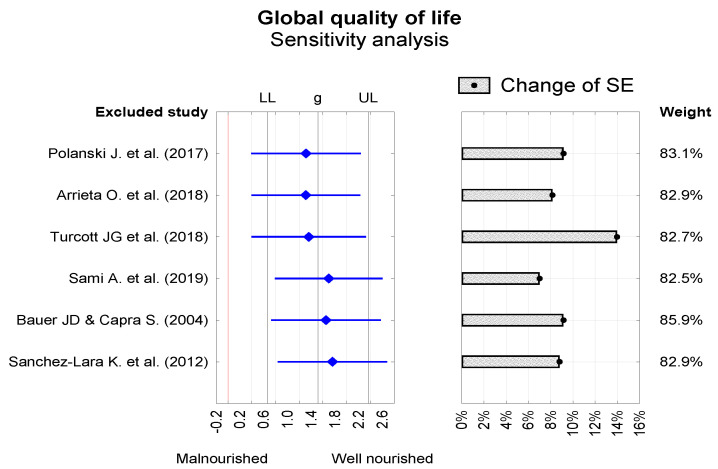
Sensitivity analysis for the impact of individual study exclusion on summary global QoL results (Hedge’s g).

Publication bias was evaluated using the trim-and-fill method and funnel plots (Figure 3). The impact of studies included in the meta-analysis on the final result was evaluated by sensitivity analysis (Figure 4).

QoL in terms of the physical, social, and role functioning domains of the EORTC QLQ-C30 was evaluated in five studies. Emotional functioning was evaluated in four studies, and cognitive functioning in only three. Clear heterogeneity was found in all of these studies. Heterogeneity analysis results are listed in Table 3.

**Figure 5:**
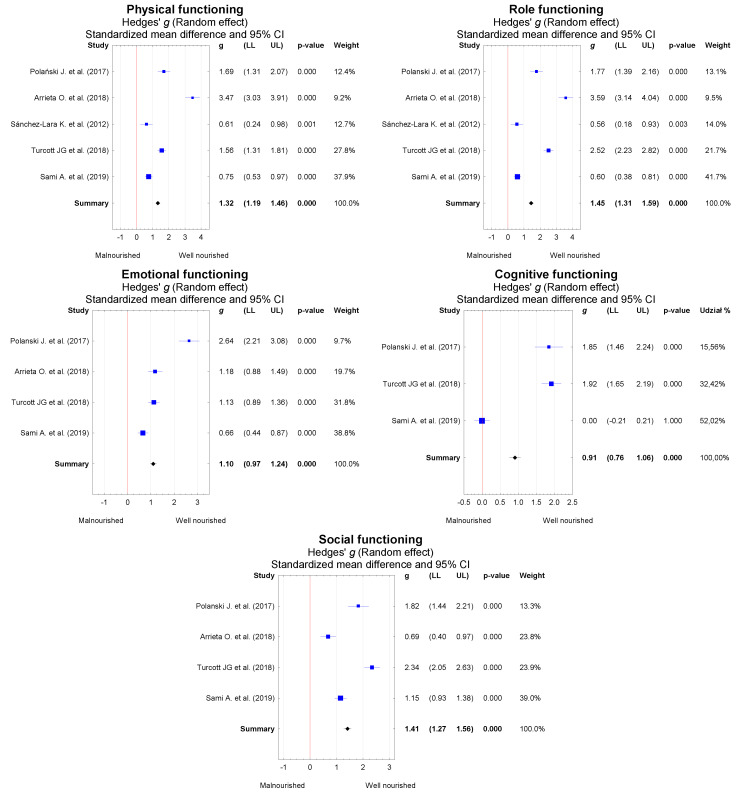
Meta-analysis results for five QoL domains in lung cancer patients broken down by nutritional status.

Due to the clear heterogeneity of the studies included in the meta-analysis, random effects models were applied. Our findings, shown in forest graphs (Figure 5), demonstrate that patients with a good nutritional status have a better QoL than malnourished patients in the following functioning domains: physical (*g* = 1.22, 95% CI = 1.19 to 1.46, *p* < 0.001), role (*g* = 1.45, 95% CI = 1.31 to 1.59, *p* < 0.001), emotional (*g* = 1.10, 95% CI = 0.97 to 1.24, *p* < 0.001), cognitive (*g* = 0.91, 95% CI = 0.76 to 1.06, *p* < 0.001), and social (*g* = 1.41, 95% CI = 1.27 to 1.56, *p* < 0.001).

### 3.3. Nutritional Status and Overall Survival (OS)

**Figure 6:**
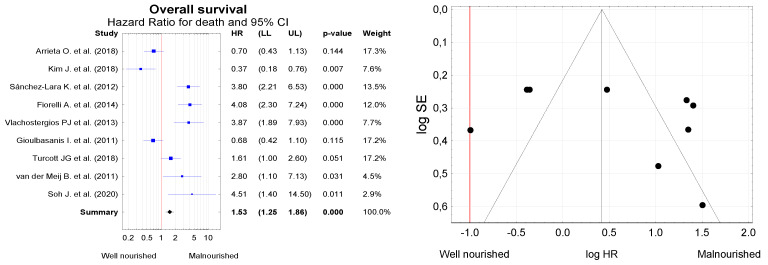
Risk of death in lung cancer patients differing by nutritional status (expressed by hazard ratio (HR)), and funnel plot for visual assessment of the probability of systematic bias due to selective publication of studies. LL—lower limit of the confidence interval; UL—upper limit of the confidence interval for the effect measure (*HR*).

The meta-analysis included nine papers providing data on the studied lung cancer patients’ overall survival (OS) and nutritional status (Figure 6). In total, the analysis included 1560 patients aged 64.6 ± 16.3 years, of whom 1064 (68.2%) were male. Based on the available data on OS, we estimated the summary hazard ratio (HR). The Kaplan–Meier survival analysis and Cox proportional hazards models were used to investigate the effect of nutritional status on survival. In these nine studies, clear heterogeneity was identified (Q = 69.6, df = 8, *p* < 0.001; I^2^ = 93.3%), and therefore, the random effect model was used for the analysis. The heterogeneity of the observed values may result, among other factors, from differences in sampling, e.g., in terms of disease stage, which is why the conclusion that malnourished patients are at more risk of death should be treated with caution. The asymmetrical concentration of studies in the funnel plot (Figure 5) indicates a possible systematic publication bias. However, summary results suggest that the risk of death is indeed significantly higher in malnourished than in well-nourished patients (*HR* = 1.53, 95% CI = 1.25 to 1.86, *p* < 0.001). 

### 3.4. Nutritional Status and Time to Tumor Progression (TTP)

**Figure 7:**
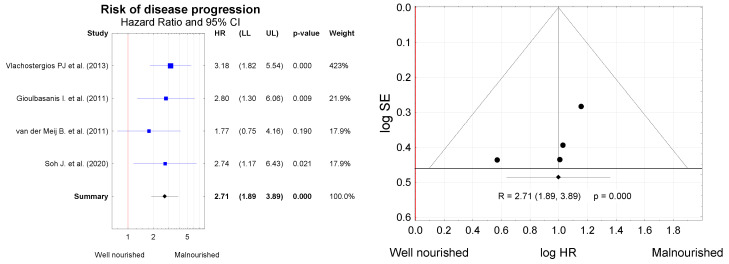
Risk of disease progression in lung cancer patients differing by nutritional status (expressed by hazard ratio (HR)), and funnel plot for visual assessment of the probability of systematic bias due to selective publication of studies. LL—lower limit of the confidence interval; UL—upper limit of the confidence interval for the effect measure (*HR*).

The meta-analysis included four papers providing data on time to tumor progression (TTP) and nutritional status in the studied lung cancer patient groups (Figure 7). Based on the available data on TTP, we estimated the summary hazard ratio (HR). The Kaplan–Meier survival analysis and Cox proportional hazards models were used to investigate the effect of nutritional status on survival. In total, the analysis included 395 patients aged 64.2 ± 15.8 years, of whom 319 (80.7%) were male. In these four studies, clear homogeneity was identified (Q = 1.28, df = 3, *p* < 0.734; I^2^ = 0.0%), and therefore, the fixed effects model was used for the analysis. Meta-analysis results warrant the conclusion that nutritional status is significantly associated with survival, as patients with a poorer nutritional status were at more risk of cancer relapse. 

## 4. Discussion

The present meta-analysis is the first one to address the important matter of associations between nutritional status and overall survival, time to tumor progression, and QoL in patients treated for lung cancer. This shows that published studies comprehensively assessing patients’ nutritional status, clinical parameters, and QoL are scarce. The present findings suggest that the introduction of standards for nutritional status and clinical condition assessment in lung cancer patients should be considered.

Lung cancer and its treatment have an impact on patients’ nutritional status, as they affect their metabolism and contribute to reduced food intake. Research demonstrates that malnutrition is a predictor of morbidity, length of hospitalization, complications, and adverse events, and in patients with advanced cancers, it is a major factor in perioperative risk assessment [9]. Thus, malnutrition may affect the duration of hospitalization, incidence of cancer recurrences, and QoL of cancer patients [12,17,18,19]. Identifying and treating nutritional problems in this patient group may contribute to better prognosis and response to therapy and reduce complications associated with the disease and its treatment [31].

The present meta-analysis affirms the conclusion that QoL is associated with nutritional status [12,17,18,19]. Its results suggest a significant difference in global QoL between well-nourished and malnourished patients, with poorer QoL in the latter group. Similar findings concern the specific EORTC QLQ-C30 domains. Malnourished patients had poorer QoL in the physical, emotional, cognitive, social, and role functioning domains. 

Progressive weight loss often reduces physical fitness and QoL in patients with advanced lung cancer [17]. Available studies indicate a better QoL in well-nourished than in malnourished patients, especially in the “role functioning” domain, with a difference of 41.6 points. In Arrieta et al., FAACT scales were significantly associated with clinical parameters, including biochemical and nutritional variables, and strongly correlated with the appetite loss subscale of the QLQ-C30 questionnaire (*r* = −0.624) [6]. In addition, Gupta et al. emphasized that normally nourished patients had significantly lower symptom severity scores than those with malnutrition [32]. 

Malnutrition is an often-unrecognized and underestimated factor in the evaluation of morbidity and mortality risk. Few studies have as yet addressed the issue. The present meta-analysis shows that malnutrition is a risk factor for complications and exacerbations (relapse). In the study by Gioulbasanis et al., MNA classification was significantly associated with time to tumor progression in patients exposed to systemic therapy, and with OS in all accrued patients [11]. Malnourished patients are at more risk of death than normally nourished ones [7,8,9,10]. A higher baseline albumin level is positively correlated with survival [7,8]. Arrieta et al. showed that physical well-being (*p* < 0.0009), functional well-being (*p* = 0.004), and the anorexia/cachexia scale score (*p* = 0.029) were all strongly associated with overall survival [6]. Similarly, our meta-analysis shows shorter survival in malnourished patients than in those with a normal body weight. Jagos et al. documented a significant relationship between low serum albumin levels and low BMI with increased postoperative morbidity (major infection) and respiratory mortality, and in the present meta-analysis, nutritional assessment was based on the MNA and SGA scales [33]. Soh et al. demonstrated that the prognostic nutrition index (PNI) decreased significantly as treatment progressed [14]. Patients at clinical stage cT3/4 had a significantly lower PNI than those with cT1/2, whereas the extent of lymph node metastasis did not affect PNI. Moreover, high PNI before ICRT significantly correlated with better survival in patients with a locally advanced NSCLC, especially at clinical stage cT3/4 [14]. In the study by Turcott et al., BMI (<18.5 vs. 18.5–24.9 vs. 25 kg/m^2^) and the presence of anorexia were shown to be independently associated with OS [12]. 

According to the European Society of Parental and Enteral Nutrition (ESPEN) guidelines, malnutrition is diagnosed in patients with BMI <19.8, albumin levels <3.0, and transferrin levels <1.5, but in elderly lung cancer patients undergoing surgical treatment, cardiorespiratory function and the extent of resection should also be evaluated. In their study, Fiorelli et al. indicate that malnutrition is an additional risk factor for mortality within 1 year of the surgical intervention [9]. The authors state that nutritional support before and after surgery may provide major benefits, and in combination with multi-disciplinary care, it might facilitate a gradual physiological return to normal activity and positively influence the outcome [9]. Polański et al. showed that as few as 25% of the patients with NSCLC are normally nourished, and only these patients rate their QoL as good in all EORTC QLQ-C30 and LC-13 functioning scales, while experiencing less severe symptoms [17]. In the same paper, malnutrition correlated with poorer QoL and worse symptoms, and was an independent determinant of decreased QoL [17]. Notably, lung cancer symptoms are accompanied with poorer QoL, which is particularly affected by appetite loss, while the symptoms themselves have a major impact on appetite and food intake [17]. The authors agree that patients suffering from fever, anorexia, and weight loss are among those who do not respond to chemotherapy. Chemotherapy has no significant impact on respiratory function or nutritional status and does not improve QoL [17]. Besides the fact that a 5% weight loss in the induction period predisposed patients to shorter OS, van der Meij et al. found that the specific combination of being overweight with a 5% weight loss in the induction period was associated with both worse OS and PFS, which suggests that when malnutrition develops during induction CRT in overweight patients, it hinders both the surgical outcome and the long-term cancer outcome [13]. This is why the nutritional status of (overweight) patients undergoing CRT and surgical treatment for NSCLC should be monitored throughout the treatment period, not just at the beginning of therapy, and nutritional interventions should be an integral part of the treatment process in patients with lung cancer. The correct management model can be implemented if QoL assessment is included in clinical condition evaluation besides the monitored clinical parameters, with the obtained results continuously analyzed in terms of their impact.

In the practice of oncology, estimating baseline nutritional status by calculating percentage weight loss is an overly simplistic approach and, on the other hand, an in-depth assessment of nutritional status is impractical in many cases. An urgent and currently unmet need is to develop an accurate, practical, and non-time-consuming screening and/or assessment tool for nutritional status. MNA, SGA, or even BMI should be considered when assessing nutritional status in lung cancer patients and possibly in patients with other malignancies in which malnutrition occurs with equal frequency.

## 5. Study Limitations

Some of the studies included in the meta-analysis may not be properly blinded, as a description was missing. Though the quality of all included studies was high, nonblinded studies may introduce an inevitable systematic bias. Moreover, the criteria used to identify malnutrition in the patients differed between studies. The analyses of significant variables (QoL in specific domains, overall survival) were considerably heterogeneous. However, statistical findings did not change with the exclusion of individual studies in sensitivity analysis, which lends credibility to our conclusions. In the study, specific treatments and cancer stages were not considered as inclusion criteria. 

## 6. Conclusions

Nutritional status is a significant clinical and prognostic parameter in the assessment of lung cancer treatment. Malnutrition is associated with poorer outcome in terms of overall survival, time to tumor progression, and QoL in patients treated for lung cancer. 

## Figures and Tables

**Table 1:** Summary of studies: INI—Inflammatory-Nutritional Index; OS—overall survival; NSCLC—Non-small-cell lung carcinoma; FAACT—The Functional Assessment of Anorexia/Cachexia Therapy; SEMS—self-expandable metallic stents: PG—percutaneous gastrostomy; AD—adenocarcinoma; PNI—the prognostic nutritional index; Icrt—comprised of induction chemoradiotherapy; MNA—mini-nutritional assessment; SGA—Subjective Global Assessment; BMI—body mass index; AC/S—anorexia-cachexia subscale; TTP—time-to-tumor progression; LOS—length of hospital stay.

No.	Author and Year	Study Group	Study Design	Outcome	Main Results and Coclusions	Intervention
1	Polański J et al., 2017 [17]	180 patients with NSCLC aged 62.8 ± 9.6 years Zubrod score: 0–18.1%; 1–37.5%; 2–36.1%; 3–4.2%; 4–0.5%	cross-sectional observational study	quality of life	The multivariate analysis revealed that nutritional status is an independent determinant of diminishing quality of life within the physical functioning scale (β = −0.17; *p* = 0.001), and of increasing severity of nausea and vomiting (β = 0.005, *p* = 0.009) and insomnia (β = 0.003, *p* = 0.011) within the symptom scales.	NO
2	Arrieta O et al., 2018 [6]	200 patients (84 male) aged 61.8 ± 13.2 years 67% adenocarcinoma, 14% squamous cell carcinoma In 78% of patients, the clinical stage was IV, in 13% stage III, and 3% stage I or II.	validation study	overall survival	The median post-questionnaire survival was 10.7 months. FAACT scales presented significant associations with clinical parameters, including biochemical and nutritional variables (i.e., energy intake, *p* = 0.002), as well as strongly correlated with the appetite loss subscale of the QLQ-C30 questionnaire (*r* = −0.624). Physical well-being (*p* < 0.0009), functional well-being (*p* = 0.004), anorexia/cachexia scale (*p* = 0.029), and FAACT total scores (*p* = 0.0009) were strongly associated to overall survival.	NO
3	Kim J et al., 2018 [7]	84 patients with lung cancer who underwent either SEMS insertion (stent group—68) or PG (gastrostomy group—16) as an initial treatment procedure for dysphagia	comparative Study	overall survival	Multivariate analysis revealed a higher baseline albumin level to be positively related to a better survival.	NO
4	S´anchez-Lara K et al., 2012 [8]	119 (64 male) patients with NSCLC aged 60.5 ± 12.5 years. 27.7% stage IIIB and 72.3% stage IV.	prospective study	overall survival	OS at 12 month in the low-risk group of malnutrition was 78.4% (95% CI, 72.1–84.7%), although this was 53% (95% CI, 47.1–58.8%) in the intermediate-risk group and 13.8% (95% CI: 9.04–18.5%) in the high-risk group. W analizie wieloczynnikowej malnutrition was an independent predictor of OS (95% CI, 1.3–15.5; *p* = 0.005).	NO
5	Fiorelli A et al., 2014 [9]	94 patients aged 74.9 ± 2.6 years (94 male) 35% squamous cell carcinoma and 51% adenocarcinoma. Stage: Ia 20.5%; Ib 32.5%; IIa 5.1%; IIb 23.1%; IIIa 13.7%; IIIb 3.4%; IV 1.7%	retrospective study	mortality, overall survival	On multivariate analysis, significant risk factors for early mortality was weight loss (*p* = 0.007). BMI of less than 18.5 (*p* = 0.01) and weight loss of >5% before operation (*p* = 0.01) were independent risk factors for 1 year mortality. Patients with weight loss >5% had a significantly worse overall survival than control group in the first 13 months after an operation (*p* = 0.01).	NO
6	Vlachostergios PJ et al., 2013 [10]	103 patients with NSCLC (93 male) aged 67 (32–84) years. 77.6 % received systemie antineoplastic therapy.	longitudinal cohort study	mortality, overall survival, TTP, LOS	W analizie wieloczynnikowej albuminy były istotnym statystycznie determinantem progression-free survival, a MNA i albuminy były niezależnymi czynnikami prognostycznymi overall survival.	lipopolysaccharide (LPS)-stimulation
7	Gioulbasanis I et al., 2011 [11]	114 patients with NSCLC aged 67.5 ± 5.4 years. 100% stage IV, 72.8% adenocarcinoma, 27.2% nonadenocarcinoma, 21.1% squamous cell carcinoma.	evaluation study	TTP, overall survival	Univariate analysis revealed that MNA classification was significantly associated with TTP in patients exposed to systemic therapy and OS in all accrued patients, co zostało potwierdzone również in multivariate analysis (TTP: Group A vs. B: HR = 2.348; *p* = 0.018 and Group A vs. C: HR = 3.427; *p* = 0.004; OS: Group A vs. B: HR = 3.273; *p* < 0.001) and Group A vs. C: HR = 4.694; *p* < 0.001).	NO
8	Turcott JG et al., 2018 [12]	312 patients with NSCLC (137 male) aged 60.6 ± 14.1 years. 81.8% presented with stage IV disease, while 18.2% had stage III. Additionally, 67% had adenocarcinoma histology and most patients (46.4%) were undergoing first-line chemotherapy.	cross-sectional study	quality of life, overall survival	In the multivariate analysis body mass index [(<18.5 vs. 18.5–24.9 vs. 25 kg/m^2^) and the presence of anorexia using the A/CS scale [(32 vs. >32); HR: 1.6 (95% CI: 1.0–2.6; p¼0.045)] were shown to be independently associated with OS.	NO
9	van der Meij BS et al., 2011 [13]	51 patients (26 male) NSCLC aged 57(39–74) years. 26 patients with involvement of N2/N3 lymph nodes and 41 patients with a SST or T4 tumor.	retrospective study	TTP, overall survival, PFS	Weight loss 5% from baseline until surgery was associated with shorter OS (HR 2.80, *p* = 0.03). Especially overweight patients who experienced a weight loss of 5% tended to have a shorter OS (adjusted HR 4.63, *p* = 0.005) and progression-free survival (adjusted HR 6.03, *p* = 0.007).	NO
10	Soh J et al., 2020 [14]	127 patients (99 male) aged 61 (31–79) years. Clinical stage: T1—19, T2—35, T3—32, T4—40. Histological subtype: 70 AD and 57 others.	retrospective study	TTP, overall survival	Multivariable analyses revealed that a high PNI pre-iCRT correlated significantly with a better survival of NSCLC patients, especially those with cT3/4 disease (hazard ratio 3.84; 95% confidential interval 1.34–12.5, *p* = 0.012).	NO
11	Antoun S et al., 2019 [18]	531 patients (353 male) aged 65.2 ± 10.0 years. The tumour stage: I–II: 34, IIIA: 21, IIIB–IV: 440, unknown 9. Histological subtype: 140 squamous cell carcinoma, 348 adenocarcinoma 18 large cell carcinoma, 25 other.	cross-sectional and non-interventional multicentre study	quality of life	The more advanced the cachexia stage, the poorer the scores of functional items of the QoL (*p* < 0.001). The presence of anorexia was associated with more advanced cachexia stages: AC/C (*p* < 0.0001) and QLQ-C30 (*p* < 0.0001). The functional score (except for cognitive) of the QoL questionnaire decreased significantly with advanced cachexia stages (*p* < 0.001).	NO
12	Bauer JD and Capra S, 2005 [19]	7 patients (5 male) aged 55.1 ± 5.0 years including 2 patients with lung cancer	cohort study	quality of life	Change in nutritional status as determined by PG-SGA score was significantly associated with change in quality of life (*r* = −0.835, *p* = 0.020) and change in lean body mass (*r* = −0.998, *p* = 0.040).	NO

**Table 2:** Methods for assessing the nutritional status and quality of life used in the included studies.

Study	Nutritional Status Measure	Outcome Measure
[17]	MNA	EORTC QLQ-C30
[6]	BMI, SGA, AC/S	OS
[7]	BMI, albumin	OS
[8]	BMI, albumin, SGA	OS
[9]	BMI	number of deaths, OS
[10]	BMI, albumin, MNA	TTP, number of deaths, LOS, OS
[11]	BMI, MNA	TTP, OS
[12]	AC/S	EORTC QLQ-C30, OS
[13]	WL ≤ 5%	TTP, OS
[14]	BMI	TTP, OS
[18]	CAX	EORTC QLQ-C30
[19]	SGA	EORTC QLQ-C30

OS—overall survival; MNA—Mini Nutritional Assessment; SGA—subjective global assessment; BMI—body mass index; AC/S—anorexia–cachexia subscale; CAX—cachexia; WL—weight loss; TTP—time to tumor progression; LOS—length of hospital stay.

**Table 3:** Study heterogeneity results for the analyzed QoL domains.

Quality of Life Domains	Q	df	*p*	I^2^
Global quality of life	99.3	4	<0.001	96.0%
Physical functioning	139.2	4	<0.001	97.1%
Role functioning	223.2	4	<0.001	98.2%
Emotional functioning	65.3	3	<0.001	95.4%
Cognitive functioning	148.4	2	<0.001	98.6%
Social functioning	74.0	3	<0.001	95.9%

## Data Availability

The data presented in this study are available on request from the corresponding author.
